# Characteristics of a Microcystin-Degrading Bacterium under Alkaline Environmental Conditions

**DOI:** 10.1155/2009/954291

**Published:** 2010-02-22

**Authors:** Kunihiro Okano, Kazuya Shimizu, Yukio Kawauchi, Hideaki Maseda, Motoo Utsumi, Zhenya Zhang, Brett A. Neilan, Norio Sugiura

**Affiliations:** ^1^Graduate School of Life and Environmental Sciences, University of Tsukuba, 1-1-1 Tennodai, Tsukuba, Ibaraki 305-8572, Japan; ^2^Graduate School of Bioresource Science, Akita Prefectural University, 241-438 Kaidobata-Nishi, Nakano Shimoshinjo, Akita 010-0195, Japan; ^3^Mitsubishi Chemical Analytech Co. Ltd., 8-5-1 Tyuuou, Ami, Inashikigun, Ibaraki 300-0332, Japan; ^4^Faculty of Engineering, The University of Tokushima, 2-1 Minamijosanjima-cho, Tokushima, Tokushima 770-8506, Japan; ^5^School of Biotechnology and Biomolecular Sciences, The University of New South Wales, Sydney, NSW 2052, Australia

## Abstract

The pH of the water associated with toxic blooms of cyanobacteria is typically in the alkaline range; however, previously only microcystin-degrading bacteria growing in neutral pH conditions have been isolated. Therefore, we sought to isolate and characterize an alkali-tolerant microcystin-degrading bacterium from a water bloom using microcystin-LR. Analysis of the 16S rRNA gene sequence revealed that the isolated bacterium belonged to the genus *Sphingopyxis*, and the strain was named C-1. *Sphingopyxis* sp. C-1 can grow; at pH 11.0; however, the optimum pH for growth was pH 7.0. The microcystin degradation activity of the bacterium was the greatest between pH 6.52 and pH 8.45 but was also detected at pH 10.0. The *mlrA* homolog encoding the microcystin-degrading enzyme in the C-1 strain was conserved. We concluded that alkali-tolerant microcystin-degrading bacterium played a key role in triggering the rapid degradation of microcystin, leading to the disappearance of toxic water blooms in aquatic environments.

## 1. Introduction

Toxic blooms of cyanobacteria frequently occur in eutrophic lakes, ponds, and reservoirs throughout the world [1–7]. It has been reported that up to 70% of these blooms are potentially toxic [8]. Cyanobacteria, such as *Microcystis*, *Anabaena*, *Planktothrix* (*Oscillatoria*), *Nostoc*, and *Hapalosiphon* species, produce a family of cyclic heptapeptide hepatotoxins called microcystins (MCs) [9, 10]. The most common MC is microcystin-LR (MCLR) that has the structure cyclo (D-Ala-L-Leu-D-MeAsp-L-Arg-Adda-D-Glu-Mdha), where MeAsp stands for D-erythro-*β*-methylaspartic acid and Mdha, for *N*-methyl-dehydroalanine. Adda is 2*S*, 3*S*, 8*S*, 9*S*-3-amino-9-methoxy-2, 6, 8-trimetyl-10-phenyldeca-4*E*, 6*E*-dienoic acid [9]. 

MCs inhibit protein serine/threonine phosphatases 1 and 2A and promote tumour growth [10–13]. An important concern is that chronic exposure to low concentrations of MCs in drinking water may promote tumour growth in the human liver [11, 14]. Currently, drinking water supplies all over the world are regularly contaminated with MCs, posing a threat to public health. For example, in 1996, more than 50 haemodialysis patients in Caruaru, Brazil, died because the water used for dialysis was contaminated with MCs [15, 16].

In the aquatic environment, the dynamics of MCs typically depend on the population density of *Microcystis* sp. [1, 2, 5–7]. Levels of MCs in water have been observed to dramatically decrease with any reduction in the population density of *Microcystis* that occurs during the disintegration of blooms [1, 2, 5–7]. Under natural conditions, MCs are resistant to physicochemical and biological stresses, such as pH, temperature, sunlight, and certain enzymes [17–20]. Biodegradation trials of MCs have been conducted [21, 22]; however, Okano et al. reported that common enzymes, including proteases, cannot degrade MCs [20]. Therefore, it was assumed that MCs can be degraded only by specific MC-degrading bacteria. In 1994, Jones et al. isolated the first MC-degrading bacterium *Novosphingobium* sp. (synonymous with *Sphingomonas* sp.) [21]. Subsequently, ten strains of MC-degrading bacteria have been isolated and studied in detail [23–32]. Bourne et al. suggested an enzymatic pathway for the degradation of MCLR that included the proteins MlrA, MlrB, and MlrC, as well as identifying the gene cluster encoding these enzymes (*mlrA*, *mlrB*, *mlrC*, and *mlrD*) in an environmental isolate [33, 34]. It was also determined that the *mlrA* gene codes for an enzyme responsible for the hydrolytic cleavage of the cyclic structure of MCLR [33], while the *mlrD* gene forms an operon with *mlrA* and encodes a putative transporter protein of MC and its degradation products [34]. In addition, Imanishi et al. also suggested an enzymatic pathway for MC degradation in *Sphingomonas* sp. strain B9 [35]. According to the results of these previous studies, MlrA, MlrB, and MlrC probably catalyze the degradation of MCLR, linear MC, and the tetrapeptide H-Adda-Glu-Mdha-Ala-OH, respectively, with the release of Adda and oligopeptides. No information, however, is available on the mechanism underlying the dramatic degradation of MCs in natural waters. Aquatic monitoring has revealedthe high pH (8.5–11.0) of the upper layer of natural waters during the sudden proliferation of water blooms as a consequence of the photosynthetic activity of cyanobacteria [3, 5–7]. Therefore, in order to rapidly degrade MCs, a significant number of MC-degrading bacteria must be able to proliferate and show activity in the alkaline pH range. However, to date only MC-degrading bacteria growing at a neutral pH have been isolated. For example, *Novosphingobium* sp. strain MD-1 cannot grow under alkaline conditions [24].

Characterization of an MC-degrading bacterium that can grow at high pH would contribute to a better understanding of MC dynamics in aquatic environments and may also provide useful applications in the field of water quality management and public health. In this study, we have examined the characteristics of an alkaliphilic/alkali-tolerant MC-degrading bacterium under high pH environmental conditions.

## 2. Materials and Methods

### 2.1. Isolation of the MC-Degrading Bacterium

The bacterium was isolated from Hongfeng Lake in Guizhou Province, China, where cyanobacterial blooms have regularly occurred. Water samples collected from the lake water column were filtered using GF/C glass-fiber filters (mean pore diameter, 1.2 *μ*m; Whatman, Japan). To this filtered lake water, added MCLR was added to a final concentration of 2,000 *μ*g/L (Wako Pure Chemical Industries Ltd., Japan) in 0.1 M carbonate buffer (pH 9.5) and an antibacterial-antimycotic mixed stock solution (Nacalai Tesque Japan) containing final concentrations of penicillin (10 unit/mL), streptomycin (100 *μ*g/mL), and amphotericin B (0.25 *μ*g/mL). The samples were then cultured at 25°C for 7 days with shaking. This process was repeated several times to isolate the MC-degrading bacterium. To confirm whether a pure culture had been isolated, it was plated onto diluted (1/20) peptone-yeast (PY) medium (peptone, 0.5 g; yeast extract, 0.25 g; agar, 15 g; per liter, pH 7.0). Subsequently, the isolated bacterium was partially characterized by Gram-staining and named C-1. After culturing in diluted PY liquid medium for 3 days, 20 mL aliquots were placed in 2 mL serum tubes and stored at −80°C.

### 2.2. 16S rRNA Gene Analysis

The isolate was cultured on diluted PY liquid medium and recovered by centrifugation at 15000 × g for 5 minutes before DNA extraction. The PrepMan Method (Applied Biosystems, CA) was used for genomic DNA extraction according to the manufacturer's instructions. PCR was performed using Ex *Taq* DNA polymerase (Takara Bio Inc., Shiga, Japan) and the 27F-1492R primer set for amplification. Cycle sequencing of the amplified 16S rRNA gene was performed using the ABI Prism BigDye Terminator v. 3.0 Ready Reaction Cycle Sequencing Kit (Applied Biosystems), according to the manufacturer's protocol. Purification of amplified and cycle sequencing PCR products utilized a MicroSeqFull 16S rRNA gene bacterial sequencing kit (Applied Biosystems) according to the manufacturer's protocol. 

Sequence data were assembled using the AutoAssembler 2.1 (Applied Biosystems) in order to determine the 16S rRNA gene sequence (GenBank accession number AB161684) and used in a BLAST search (http://www.ncbi.nlm.nih.gov/BLAST) to retrieve homologous sequences from GenBank (http://www.ncbi.nlm.nih.gov/Genbank). The 16S rRNA gene sequences of related species obtained by the BLAST search together with those of other MC-degrading bacteria were aligned using Clustal X version 2.0 [36]. The data were used for neighbor-joining analysis with 1000 bootstrap replicates, using the NJ plot program [37] and the phylogenetic tree was reconstructed.

### 2.3. Identification of the mlrA Gene and Sequence Analysis

A FastDNA kit (Qbiogene Inc., USA) was used to extract the genomic DNA of strain C-1, according to the manufacturer's instructions. Presence of the *mlrA* gene was confirmed by PCR amplification as per Saito et al. [38]. The PCR was carried out using the MF (5′-GACCCGATGTTCAAGATGCT-3′) and MR (5′-CTCCTCCCACAAATCAGGAC-3′) primers and KOD Plus polymerase (Toyobo, Japan). The amplified fragments (approximately 800 bp) were purified using a QIAquick PCR purification kit (Qiagen KK, Japan), and then cycle sequencing was performed using the ABI Prism cycle sequencing kit. The DNA sequence was edited and aligned using GENETYX-MAC (Software Development, Japan), and a BLAST search (http://www.ncbi.nlm.nih.gov/BLAST) was performed against homologous sequences in GenBank (http://www.ncbi.nlm.nih.gov/Genbank). The sequence of the *mlrA-*C-1 gene was registered in GenBank with the accession number AB161685.

### 2.4. Effect of pH on Growth

After isolating the newly isolated MC-degrading bacterium (strain C-1) along with *Novosphingobium* sp. strain MD-1, isolated from Lake Kasumigaura, on 1.5% agar plates containing PY medium (peptone, 10 g; yeast extract, 5 g; pH 7.0), the cells were precultured at 28°C for approximately 24 hours in test tubes containing liquid PY medium. A 1/10 volume of bacterial culture in the late logarithmic growth phase was inoculated into a series of liquid PY media with pHs ranging from 7.00 to 11.0, (adjusted by using 1 M NaOH). The optical density of the solutions was measured at 600 nm every 2 hours by using the GeneQuant Pro spectrophotometer (GE Healthcare Bio-Sciences AB, Sweden).

### 2.5. Effect of pH on MC Degradation Activity

Strain C-1 was precultured in 150 mL of diluted (1/20) PY medium (pH 9.5) in a 500 mL flask and collected by centrifugation at 3000 × g for 5 minutes, followed by washing the pellet with sterilized milliQ water. This procedure was repeated three times, and the resultant pellet was used as the sample. The sample was inoculated into test tubes containing microcystin medium (1000 *μ*g/L MCLR, 0.1 M phosphate buffer; pH 7.0) with an optical density adjusted to 0.5 at 600 nm and then cultured at 25°C for 3 hours with shaking. The final volume of the medium was 5 mL, and the MCLR concentration was measured at 0 hour and 3 hours. The pH of the MC medium was varied from 6.29, 6.72, 7.06, 7.48, 7.92, 8.04, 8.45, 8.91, 9.5, and 10.2 by using either 0.1 M phosphate buffer or 0.1 M borate buffer. The experiment was repeated three times, and the standard deviations and means were evaluated for each treatment.

### 2.6. Analysis of MC and Its Primary Degradation Products

MC was quantitatively analyzed using the Shimadzu 10A series HPLC system (Shimadzu Corporation, Japan) and a Cosmosil C18 column (4.6 × 250 mm; Nacalai Tesque). Methanol (60%) diluted with 0.05 M phosphate buffer (pH 3.0) was used as the mobile phase, and the flow rate was set at 1.0 mL/min. The analysis was conducted at a column temperature of 40°C and using a detection wavelength of 238 nm.

The culture samples of strain C-1 were inoculated into MC medium containing MCLR (3000 *μ*g/L), and the final optical density of the medium was adjusted to be 5.0 at 600 nm. The culture was then incubated at 25°C with shaking. Once the degradation of MC had been confirmed, the bacterial culture was separated by centrifugation at 3000 ×g for 5 minutes and the resultant bacterial pellets were collected. The cells were resuspended in phosphate buffer, sonicated using the ultrasonicator (VC-70, Sonics & Materials, Inc., USA), and separated by centrifugation at 15,000 × g for 10 minutes. The supernatant was filtered using 0.2 *μ*m filters (HPLC Millex, Millipore, Japan) to obtain the cell-free extract (CFE) of strain C-1. Four milliliters of the CFE were mixed with MCLR to a final concentration of 3000 *μ*g/L. The pH of the solution was adjusted to 7.5 using 50 mM phosphate buffer, and the degradation reaction was initiated at 25°C. The reaction volume was 5 mL, and 0.1% sodium azide was added for sterilization of the solution. MCLR were treated with the CFE before purification, using a Sep-Pak C_18_ plus cartridge (Waters, USA). Phenylmethylsulfonyl fluoride (10 mM) and ethylenediaminetetraacetic acid (1 mM) were added to the reaction solution to inhibit serinegand metalloproteases since these inhibitors do not affect MlrA [33]. The purified samples were analyzed by high-performance liquid chromatography (Agilent 1100 Series; Agilent Technologies Inc., CA) and mass spectrometry (API300; Applied Biosystems) using an Inertsil ODS-3 column (2.1 × 150 mm; particle size, 3.0 *μ*m; GL Sciences, Japan) at 40°C. The mobile phase under linear gradient conditions was a combination of methanol (LC/MS grade; Wako Pure Chemical Industries) and 0.05% trifluoroacetic acid (TFA) (LC grade; Wako Pure Chemical Industries). The analytical time was 30 min, and the linear gradient was 0%–100% of methanol and 100%–0% of TFA (0.05%). The flow rate was set at 0.2 mL/min, and mass spectrometry analysis was conducted at an infusion rate of 10 *μ*L/min. Formic acid was added to the samples to facilitate ionization at the time of injection. Furthermore, a turbo ion spray interface and positive mode electrospray ionization (ESI) were used. The ESI conditions in positive ion mode were as follows: the interface, turbo ion spray; Ionization, electrospray ionization; the nebulizer gas and the flow rate, N_2_ and 6 L/min; the curtain gas and flow rate, N_2_ and 10 L/min; the ion spray voltage, 5000 V; the orifice voltage, and 20 V. MS data were analyzed using MacQuan software version 1.6 (Sciex, Concord, Canada).

## 3. Results

### 3.1. Isolation and Characterization

**Figure 1:**
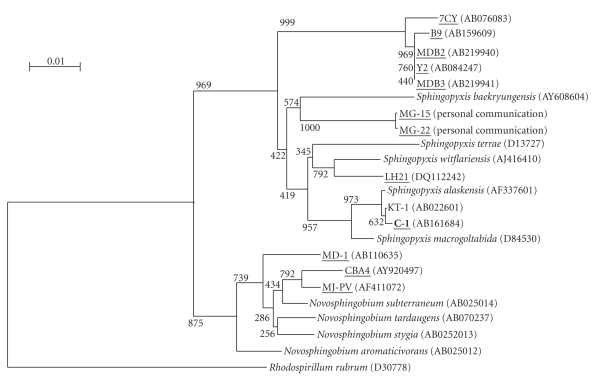
Neighbor-joining phylogenetic tree based on 16S rRNA gene sequences indicating the position of isolate C-1 (in bold type) in relation to other related species, including previously isolated MC-degrading bacteria (underlined). Numerical values represent bootstrap support. Scale bar represents expected changes per site.

The MC degrading bacterium, termed the C-1 strain, was isolated from Lake Hongfeng in Guizhou Province, China, by enrichment culture using MCLR as the major carbon and nitrogen source. The C-1 strain is Gram negative, rod shaped, and forms yellow colonies on solid PY media. Analysis of its 16S rRNA gene sequence revealed that the bacterium belonged to the genus *Sphingopyxis*, order Sphingomonadales of the *α*-proteobacteria (Figure 1). The 16S rRNA gene of strain C-1 was 99.4% similar to that of *Sphingopyxis* sp. strain KT-1 (JCM10459; accession number AB022601), which does not possess MC-degradation activity. Only the LH21 strain of *Sphingopyxis* spp. has been reported to degrade MC, while other MC-degrading strains of bacteria belong to various other genera.

### 3.2. Effect of pH on Growth and MC Degradation

**Figure 2:**
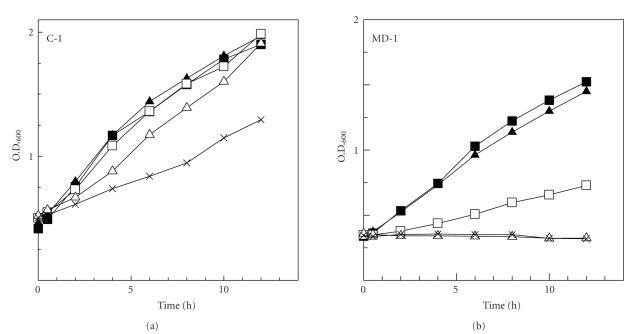
Effect of pH on growth of MC-degrading bacteria. (a) *Sphingopyxis* sp. strain C-1, (b) *Novosphingobium* sp. strain MD-1. Values presented are the means of 3 replicates. ■: pH 7.00, ▲: pH 8.00, □: pH 9.00, *∆*: pH, 10.0, ×: pH11.0.

Strain C-1 was isolated from lake water which exhibited alkaline conditions (pH 9.50). The effect of pH on the growth of the MC degrading strains C-1 and MD-1 was compared in a batch culture experiment. The growth of the MD-1 strain was inhibited significantly under alkaline conditions with an initial pH as low as 9.00 or 10.0, whereas the growth of the C-1 strain was not inhibited at a pH of 11.0 (Figure 2). However, the optimum pH for the growth of the C-1 strain was determined to be 7.00 and, therefore, the C-1 strain should be described as alkali tolerant. 

**Figure 3:**
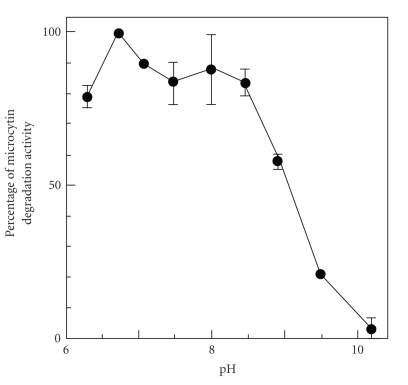
Effect of pH on microcystin-LR degradation activity of *Sphingopyxis* sp. strain C-1. MC degradation activity was determined as the MCLR concentration at 0 hour and 3 hours. The error bars indicate standard deviation (*n* = 3).

The pH of most water bodies changes dynamically during the occurrence of a water bloom and becomes highly alkaline. Hence, it was important to examine the effect of pH on the degradation activity of MC-degrading bacteria. Figure 3 shows that the MCLR degradation activity of strain C-1 was high, especially between pH 6.72 and 8.45. In addition, the C-1 strain retained 60% of its MCLR degradation activity at pH 8.91. In contrast, the degradation activity of the MD-1 strain was reduced by 80% at pH 8.50 [24]. The growth rate of the C-1 strain at pH 10.0 was comparable to its growth rate at near neutral pH although the optimum pH for degradation was around neutral (pH 6.72 to pH 8.45). Therefore, degradation may depend on the optimum pH for enzyme activity as opposed to the growth rate of the microorganism. The C-1 strain was also shown to degrade both MCLR and microcystin-RR (MCRR). HPLC chromatograms of MCLR and MCRR degradation were the same for strains C-1 and MD-1 at neutral pH (data not shown).

### 3.3. Analysis of the Primary Degradation Product

**Figure 4:**
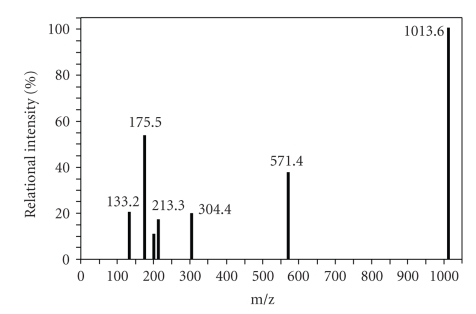
Mass spectrum of the primary degradation product of *Sphingopyxis *sp. strain C-1. The raw data obtained using mass spectrometry was treated by MacQuan and the figure shows only the main peaks.

**Figure 5:**
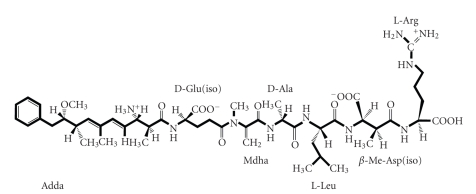
Structure of linearized microcystin-LR.

In this study, we identified an *mlrA* homolog in strain C-1 (accession number AB161685). We confirmed that the sequence of this *mlrA* homolog was highly similar to that of previously characterized *mlrA* genes from other MC-degrading bacteria. By using the LALIGN program (http://www.eng.uiowa.edu/~tscheetz/sequence-analysis/examples/LALIGN/lalign-guess.html), the predicted amino acid sequence of MlrA from strain C-1 was found to be 94.3%, 93.9%, 88.2%, and 95.9% similar to MlrA-MJ-PV (ACM 3962; AF411068), MlrA-MD-1 (AB114202), MlrA-Y2 (JCM 13185^T^; AB114203), and MlrA-LH21 (DQ112243), respectively. It was evident from these data that *mlrA* is highly conserved in all known MC-degrading bacteria from a variety of genera. Furthermore, the HEXXH motif, required for the activation of zinc protease, was also found to be highly conserved [34]. On the basis of these findings, we propose that the function of MlrA in the C-1 strain was similar to its function in the MJ-PV strain [33] and, therefore, the primary degradation products of MCLR were analyzed by mass spectrometry. The mass spectrum obtained was highly similar to that reported by Bourne et al. [33] (Figure 4). The parent and daughter ions of the degradation products are shown in Table 1. The primary degradation product was found to be linearized MCLR (Adda-Glu-Mdha-Ala-Leu-Masp-Arg-OH), the same intermediate product mentioned in previous reports of the enzymatic degradation of MC by heterotrophic bacteria isolated from water blooms [25, 33] (Figure 5).

## 4. Discussion

A dynamic change in the pH of aquatic environments from neutral to alkaline is associated with the occurrence of the blooms of cyanobacteria in water bodies [3, 5–7]. However, all currently known MC-degrading bacteria have been isolated under neutral pH conditions [21, 23–31]. Hence, the mechanism which accounts for the rapid degradation of MCs in aquatic environments remains unclear. In this study, it was assumed that alkali tolerant and/or alkaliphilic MC-degrading bacteria contribute significantly in the rapid degradation of MCs in natural waters, leading to the disappearance of water blooms and their toxic metabolites. Therefore, an alkali-tolerant MC-degrading bacterium, named C-1, was selectively isolated under alkaline conditions from a water bloom sample (Figure 2). The C-1 strain could effectively degrade MCs even in high pH to neutral conditions (Figure 3). From these results, it was considered that C-1 strain might be actively involved in the dramatic degradation of MCs at the disappearance of water blooms. Recently, Fujimoto et al. isolated strains MG-15 and MG-22 of *Monas guttula* [30] that predate on the MC-producing species *Microcystis viridis* [39]. Although these strains could not completely degrade MCLR, MCRR, and microcystin-YR, they could degrade MCs under alkaline conditions (pH 10.0). The MG-15 and MG-22 strains are also alkali-tolerant, and may also be involved in the rapid degradation of MCs since *M*. *guttula* seems to also contribute to the disappearance of water blooms [39]. 

MCs are produced and retained within cyanobacterial cells; however, approximately 10%–20% of MCs are lost from cultured cells [40, 41]. When a toxic water bloom (producing MCs) decays, MCs are released into the water column under alkaline pH conditions [42]. These MCs may then be trapped in cyanobacterial mucilage due to its high viscosity and entrapment of the cellular membranes. In fact, Maruyama et al. reported that *Sphingosinicella microcystinivorans* strain Y2, another MC-degrading bacterium, was found only in a specific region of the mucilage of *Microcystis* and the concentration of this bacterium correspondeds with the concentration of cell-bound MCs [43]. Therefore, we propose that *Sphingopyxis* sp. strain C-1 as well as other alkali-tolerant MC-degrading bacteria that inhabit the mucilage play a critical role in the degradation of MCs under alkaline pH conditions and that this contributes to bloom disintegration.

The HPLC chromatogram of MCLR degradation by C-1 strain (Figures 4 and 5) was practically identical to the chromatogram of MCLR degraded by the strains MJ-PV and B9 [33, 35]. Therefore, we hypothesize that the degradation of MCs by strain C-1 and other previously isolated MC-degrading bacteria proceeds via the same mechanism, and that the expression of MlrA initiates bacterial MC degradation. In the future it will be important not only to obtain a detailed understanding of the fate of MCs at in situ level, specifically for risk assessment, but also to analyze the mechanism of MC degradation by enzymes encoded by the *mlr* and homologous gene clusters.

## Figures and Tables

**Table 1:** Parent and daughter ions of the primary degradation product.

m/z	Identity
1013.6	M + H (Adda-Glu-Mdha-Ala-Leu-Masp-Arg-OH + 2H)
571.4	Mdha-Ala-Leu-Masp-Arg-OH + 2H
304.4	Masp-Arg-OH + 2H
213.3	Glu-Mdha + H
175.5	Arg-OH + 2H
132.2	PhCH_2_CHOCH

*Adda is 3-amino-9-methoxy-2, 6, 8-trimethyl-10-phenyldeca-4, 6-dienoic acid. Mdha is *N*-methyldehydroalanine, and Masp is D-*β*-methylaspartic acid.
